# Maternal Pregnancy-Related Anxiety Is Associated With Sexually Dimorphic Alterations in Amygdala Volume in 4-Year-Old Children

**DOI:** 10.3389/fnbeh.2019.00175

**Published:** 2019-08-06

**Authors:** Henriette Acosta, Jetro J. Tuulari, Noora M. Scheinin, Niloofar Hashempour, Olli Rajasilta, Tuomas I. Lavonius, Juho Pelto, Virva Saunavaara, Riitta Parkkola, Tuire Lähdesmäki, Linnea Karlsson, Hasse Karlsson

**Affiliations:** ^1^The FinnBrain Birth Cohort Study, Turku Brain and Mind Center, Department of Clinical Medicine, University of Turku, Turku, Finland; ^2^Department of Psychiatry, Turku University Hospital, University of Turku, Turku, Finland; ^3^Turku Collegium for Science and Medicine, University of Turku, Turku, Finland; ^4^Department of Medical Physics, Turku University Hospital, Turku, Finland; ^5^Department of Radiology, Turku University Hospital, University of Turku, Turku, Finland; ^6^Department of Pediatric Neurology, Turku University Hospital, University of Turku, Turku, Finland; ^7^Department of Child Psychiatry, Turku University Hospital, University of Turku, Turku, Finland

**Keywords:** amygdale, prenatal stress, children, SDQ, VBM, brain, development, behavior

## Abstract

Prenatal stress is associated with child behavioral outcomes increasing susceptibility for psychiatric disorders in later life. Altered fetal brain development might partly mediate this association, as some studies suggest. With this study, we investigated the relation between prenatal stress, child’s brain structure and behavioral problems. The association between self-reported maternal pregnancy-related anxiety (PRAQ-R2 questionnaire, second and third trimester) and brain gray matter volume was probed in 27 4-year-old children (13 female). Voxel based morphometry was applied with an age-matched template in SPM for the whole-brain analyses, and amygdala volume was assessed with manual segmentation. Possible pre- and postnatal confounders, such as maternal depression and anxiety among others, were controlled for. Child behavioral problems were assessed with the Strength and Difficulties Questionnaire by maternal report. We found a significant interaction effect of pregnancy-related anxiety and child’s sex on child’s amygdala volume, i.e., higher pregnancy-related anxiety in the second trimester was related to significantly greater left relative amygdala volume in girls compared to boys. Further exploratory analyses yielded that both maternal pregnancy-related anxiety and child’s amygdala volume are related to child emotional and behavioral difficulties: While higher pregnancy-related anxiety was associated with more emotional symptoms, peer relationship problems and overall child difficulties, greater left amygdala volume was related to less of these child difficulties and might partly mediate sex-specific associations between pregnancy-related anxiety and child behavioral difficulties. Our data suggest that maternal prenatal distress leads to sexually dimorphic structural changes in the offspring’s limbic system and that these changes are also linked to behavioral difficulties. Our results provide further support for the notion that prenatal stress impacts child development.

## Introduction

Early child development is shaped not only by genetic, but also by environmental factors (Greenough et al., 1987; Rice et al., 2010). According to the theory of “developmental origins of health and disease,” environmental factors during development can have long-lasting programing effects on individual health and disease susceptibility (Gluckman and Hanson, 2006; Wadhwa et al., 2009; Moog et al., 2017; O’Donnell and Meaney, 2017). The intrauterine period of life constitutes a particularly sensitive time window due to the rapid development of the embryonic/fetal organism. Risk factors related to maternal health and behavior, such as maternal psychological distress or exposure to stressful life events, that affect intrauterine development, have been established. Several studies have shown that maternal distress during pregnancy shapes behavioral, neurodevelopmental and other health-related outcomes of the offspring over the lifespan. For instance, higher self-reported maternal prenatal distress was associated with more fearfulness (Henrichs et al., 2009; Nolvi et al., 2016) and higher negative affectivity (Blair et al., 2011) of infants, with emotional and behavioral problems of children (O’Connor et al., 2002, 2003; O’Donnell et al., 2014), and anxiety disorders of young adults (Capron et al., 2015) (for a review see e.g., Bock et al., 2015; Entringer et al., 2015; O’Donnell and Meaney, 2017).

Self-reported maternal prenatal stress is a heterogeneous concept and has been defined for example as experiencing a major life event, as depressive symptoms, or as general or pregnancy-related anxiety. Pregnancy-related anxiety is a distinct anxiety construct that assesses emotional and cognitive attributes of anxiety related to fetal health and loss, childbirth, body image and perceived control (Bayrampour et al., 2016). There is some evidence that maternal pregnancy-related anxiety is of higher predictive value for some birth outcomes and infant temperament than the other distress measures (Roesch et al., 2004; Kramer et al., 2009; Nolvi et al., 2016). Yet, the biological mechanisms linking maternal prenatal distress with fetal development are not fully understood. It is assumed that the maternal-placental-fetal stress physiology partly mediates the observed outcomes via glucocorticoids, serotonin and inflammation among other factors (Entringer et al., 2015; St-Pierre et al., 2016). While glucocorticoid concentrations, vasoconstriction and other stress parameters in the mother have been inconsistently linked to self-reported maternal prenatal stress (O’Donnell and Meaney, 2017), animal and human research has revealed that chronic maternal prenatal stress is associated with a downregulation (or reduced upregulation) of the placental barrier for cortisol (11β-HSD2: 11β hydroxysteroid dehydrogenase type 2) leading to a higher fetal cortisol exposure (Welberg et al., 2005; Mairesse et al., 2007; O’Donnell et al., 2012). Accordingly, fetal cortisol exposure has been positively associated with maternal pregnancy-related anxiety (Dahlerup et al., 2018). This lends support to the notion that fetal glucocorticoid exposure might convey some of the observed effects of pregnancy-related anxiety on offspring’s outcomes.

It has been proposed that the association between prenatal stress and offspring’s outcomes might also partly be mediated by stress-related effects on brain development during the fetal period. During gestation, neuronal progenitor cells form, differentiate and migrate, and important synaptic connections are established (Andersen, 2003). Glucocorticoids affect brain neurotransmitter systems and the expression of growth factors (Bock et al., 2015). Animal studies have further revealed that prenatal stress induces neuronal and synaptic alterations that are highly region-specific. With regard to brain structure the most prominent changes were observed in prefrontal and limbic areas (Bock et al., 2015).

However, there still is a dearth of studies investigating how prenatal stress in humans, and more specifically pregnancy-related anxiety, affects human brain structure and how these brain changes are linked to child outcomes. In a study of 6-to-9-year-old children, higher maternal pregnancy-related anxiety was related to reduced gray matter volume in prefrontal, temporal, occipital and cerebellar brain regions (Buss et al., 2010). With regard to maternal prenatal general anxiety and depressive symptoms, a few longitudinal studies have indicated that maternal prenatal stress is associated with structural and functional alterations in limbic, prefrontal and other cortical brain areas (Qiu et al., 2013; Rifkin-Graboi et al., 2013, 2015; Sandman et al., 2015; Lebel et al., 2016; Wen et al., 2017; Soe et al., 2018) that were (partly weakly) associated with child’s internalizing or externalizing behavior (Rifkin-Graboi et al., 2015; Sandman et al., 2015). To our knowledge, no study has so far examined associations between child behavioral outcomes and brain structural changes that are linked to pregnancy-related anxiety. However, in one of the above-mentioned longitudinal studies, higher maternal cortisol concentrations in early pregnancy were associated with greater right amygdala volume in 6-to-9-year-old girls, but not boys (Buss et al., 2012), and the amygdala volume partially mediated the association between maternal cortisol and emotional symptoms in girls. It is conceivable that pregnancy-related anxiety is linked to similar effects given its association with fetal cortisol exposure.

The results of the study by Buss et al. (2012) also highlight another interesting aspect of fetal development: There is a growing body of evidence that prenatal stress in humans leads to sexually dimorphic birth and behavioral outcomes in the offspring (Braithwaite et al., 2017, 2018) and to sexually dimorphic associations with the amygdala (Buss et al., 2012; Wen et al., 2017; Soe et al., 2018). The amygdala contains a high density of gonadal steroid and glucocorticoid receptors and displays structural sex differences (Gray and Bingaman, 1996; Rose et al., 2004). The amygdala is part of the limbic network, plays a crucial role in emotion and salience processing, social behavior, and stress physiology, and is implicated in anxiety disorders and depression (Lindquist et al., 2012; Yilmazer-Hanke, 2012). It has further been suggested that female and male fetuses might be differently affected by the timing of prenatal stress exposure during gestation, with a higher vulnerability of males for earlier exposure compared to females (Bock et al., 2015). But while the timing of stress exposure as well as the chronicity of stress during gestation are likely relevant factors for the offspring’s outcomes, results regarding the timing of stress are still inconsistent (Graignic-Philippe et al., 2014).

With this study we aimed at investigating whether maternal pregnancy-related anxiety is associated with brain structural changes in 4-year-old children. Given the results reported by Buss et al. (2010, 2012), we hypothesized that brain gray matter volume changes are observable in several cortical areas and in the amygdala, and that the effects are partly sexually dimorphic. Finally, we wanted to explore whether pregnancy-related anxiety is associated with behavioral and emotional problems at this age and whether these associations are partly mediated by observed brain structural changes. We hypothesized that volume changes in the amygdala partly mediate the association between pregnancy-related anxiety and emotional problems in girls. Since no clear picture with regard to the role of timing of stress exposure has emerged so far, we investigated pregnancy-related anxiety of the second and third trimester separately. As a proxy for the chronicity of stress exposure we additionally investigated a sum score of pregnancy-related anxiety combining the scores of both trimesters.

## Materials and Methods

### Participants

Participants were mother-child-dyads recruited from the FinnBrain Birth Cohort Study^1^ (Karlsson et al., 2018). Neuroimaging data was collected from 33 4-year-old children. The inclusion criterion was child’s age of 4 years (47–54 months). Exclusion criteria for the children were significant developmental abnormalities of major organs (e.g., heart, limbs) and sensory systems (e.g., blindness, deafness), a diagnosis of a neurodevelopmental disorder such as autism or epilepsy, need for daily medication at the time of the scan, the lifetime experience of a severe head trauma or concussion (with unconsciousness or clinical MRI scans post trauma), and other clinical investigations, all assessed by self-report from the parents. This study was carried out in accordance with the recommendations of the Ethics Committee of the South-Western Hospital District with written informed consent from all mothers. All mothers gave written informed consent in accordance with the Declaration of Helsinki. The protocol was approved by the Ethics Committee of the South-Western Hospital District.

One subject was excluded from the analyses due to a technical failure of the MRI data acquisition. Four further subjects were excluded because of low quality of the brain structural data due to motion as assessed by visual inspection. One more subject was excluded because of missing maternal questionnaire data at the second prenatal assessment point (gestational week 24). In the final sample 27 mother–child-dyads were included [mean age of children (at MRI scan time) = 50.8 months (*SD* = 1.7, range = 47.7 – 54.0), mean age of mothers (at term) = 30.2 years (*SD* = 3.9), and 14 of the children were boys (51.9%)].

### Measures and Procedures

#### Pregnancy-Related Anxiety Questionnaire

Maternal pregnancy-related anxiety was assessed with the PRAQ-R2 questionnaire (Huizink et al., 2016) in the second and third trimester of pregnancy (gestational weeks 24 and 34). The PRAQ-R2 consists of 10 items rated from 1 to 5, and is a revised version of a measure of pregnancy-related anxiety (Huizink et al., 2004, 2016). Missing items (one item per time point at maximum) were imputed with the mean value. In this study, the sum scores of each trimester (PRAQ gwk24, PRAQ gwk34) were investigated and additionally the individual sum scores of both trimesters were combined to form a total PRAQ sum score (PRAQ Sum).

#### Strength and Difficulties Questionnaire

The Strengths and Difficulties Questionnaire (SDQ) Version P4-16 (Goodman et al., 2000; Goodman, 2001) was administered to mothers when the children were 4 years old (*N* = 21, m/f = 12/9). The SDQ is a brief behavioral screening instrument for children aged 4–16 years and measures emotional and behavioral difficulties and prosocial behavior. The questionnaire contains 25 three-point items which are divided between 5 subscales: “emotional symptoms” (F1), “conduct problems” (F2), “hyperactivity/inattention” (F3), “peer relationship problems” (F4), and “prosocial behavior.” The first four subscales that measure a child’s difficulties are summed up to generate a total difficulties score (SDQ Sum). In this study we used both the first four subscales as well as their sum score. We did not include the SDQ subscale “prosocial behavior,” because we wanted to focus on child’s difficulties in accordance with aforementioned studies (e.g., Buss et al., 2012; Rifkin-Graboi et al., 2015; Sandman et al., 2015).

#### Prenatal Maternal Control Variables

To be able to control for depressive and general anxiety symptoms, the Finnish versions of the Edinburgh Postnatal Depression Scale (EPDS) (Cox et al., 1987) and of the anxiety subscale of the revised Symptom Checklist 90 (SCL-90-R) (Derogatis, 1983; Holi et al., 1998) were administered at gestational weeks (gwk) 14, 24, and 34. Individual sum scores were computed for prenatal maternal depressive symptoms (EPDS sum) and general anxiety (SCL sum) during pregnancy. Missing values (at maximum three items per time point) were imputed with the mean value of the existing ones. The following maternal variables were assessed via mothers’ self-report at gwk 14 and/or 34: maternal education, maternal age, previous miscarriages and abortions, prenatal medication, and prenatal alcohol, nicotine and illicit drug consumption. Obstetric data was retrieved from the Finnish Medical Birth Register of the National Institute for Health and Welfare^2^, and included gestational complications (diabetes: *N* = 6, hypertension: *N* = 1) and maternal prepregnancy body mass index (BMI). We dichotomized BMI (BMI < 25, BMI > = 25) given that maternal obesity has been associated with alterations in the infant brain (Pulli et al., 2018). We further dichotomized medication use (thyroxine and corticosteroids; yes/no), alcohol and/or nicotine exposure (yes/no), gestational complications (yes/no) and previous miscarriages and/or abortions (yes/no). No significant use of antidepressants or illicit drugs was reported (three missing values at the first time point: medication: *N* = 2, illicit drugs: *N* = 1). Education was trichotomized [low: high school or vocational education (9 years), middle: (career) college (12 years), high: university (+12 years)].

#### Postnatal Maternal Control Variables

Maternal postnatal depressive symptoms were assessed by use of the Edinburgh Postnatal Depression Scale (EPDS) (Cox et al., 1987) at 3 (*N* = 27) and 6 months (*N* = 23), and at 1 (*N* = 23), 2 (*N* = 18) and 4 years (*N* = 21) post-partum. Maternal postnatal anxiety was assessed using the anxiety subscale of the revised Symptom Checklist 90 (SCL-90-R) (Derogatis, 1983; Holi et al., 1998) at 3 and 6 months, and at 2 and 4 years post-partum. Individual sum scores for postnatal maternal depressive symptoms and general anxiety were created combining the scores of all postnatal time points.

#### MRI Acquisition

Magnetic resonance imaging was performed using a 3 T Philips Ingenuity TF PET/MR (Philips, Amsterdam, Netherlands) and a Sense-Head-32 channel coil. The 3D T1 turbo field echo (TFE) sequence was imaged in sagittal orientation. The Field of view was 256 × 256 mm. Data was acquired and reconstructed with 1 mm isotropic voxels. Parallel imaging was used with a SENSE factor of 2 and a flip angle of 7°. Repetition time was 8.1 ms and echo time was 3.7 ms. The sequence duration was 4 min 23 s.

#### Manual Volume Segmentation of the Amygdala

The native anatomical images were preprocessed and segmented applying the volBrain pipeline (Manjón and Coupé, 2016). The segmentation of the amygdala volumes was amended by two raters according to the segmentation protocol by Hashempour et al. (n.d.) and by use of the software ITK-SNAP (version 3.6.0)^3^ (Yushkevich et al., 2006) and MNI Display^4^, respectively {interrater reliability [ICC(2,1)](Koo and Li, 2016): ICC (right amygdala) = 0.93, ICC (left amygdala) = 0.94}. Volumes are reported both uncorrected as well as corrected for total intracranial volume (TIV; assessed by use of volBrain).

#### Whole Brain Analysis

An age-matched template (4.75 years) was created by use of the Template-O-Matic Toolbox (TOM8) which runs in SPM8 (Wilke et al., 2008). Structural images were preprocessed with the standard routines of voxel-based morphometry in SPM8 (“New Segment”)^5^. Images were bias-corrected, tissue classified and normalized into a standard stereotactic anatomical MNI-space (resulting voxel size 1.5 × 1.5 × 1.5 mm), employing high-dimensional DARTEL normalization within a unified model (Ashburner and Friston, 2005; Ashburner, 2007). The modulated gray matter images were smoothed with a Gaussian kernel of 8 mm full width at half maximum (FWHM).

We performed SPM8 whole brain full factorial analyses with a child’s sex as factor. The individual PRAQ scores were entered as a covariate of interest interacting with the factor sex. PRAQ scores at each time point and the PRAQ Sum score were investigated in three separate models. Child’s age at MRI scan time (CAM) and total intracranial volume were always entered as covariates of no interest into the models. Localization of peaks are reported as MNI-coordinates. For the anatomical localization of the structural data, probabilistic cytoarchitectonic maps according to the SPM Anatomy Toolbox (version 2.2c)^6^ (Eickhoff et al., 2005) and the Wake Forest University PickAtlas software (version 2.5.2)^7^ were used as reference. We chose a whole brain voxel-wise threshold of *p* < 0.05 family-wise error (FWE) corrected. Additionally, we report results applying a more lenient statistical threshold (*p* < 0.001 uncorrected, minimum cluster size 50 voxels, minimum *t*-value of 5) in the Supplementary Material, but in the discussion section we will focus on the FWE-corrected results.

#### Statistical Analyses

Statistical analyses of behavioral and brain volume data were performed using R 3.4.4 (R Core Team, 2016)^8^. Packages in use were “Hmisc” (Harrell, 2017), “psych” (Revelle, 2018), “nortest” (Gross and Ligges, 2015), “ggplot2” (Wickham, 2009) and “car” (Fox and Weisberg, 2011) among others.

Missing values of postnatal control variables and the SDQ were imputed by means of multiple imputation (Rubio, 1987; Van Buuren, 2018) using the package “mice” (Van Buuren and Groothuis-Oudshoorn, 2011), and the results given from these analyses are the pooled results.

Standard multiple linear regression analyses were performed to probe the association between amygdala volumes and (a) the individual PRAQ scores and (b) the interaction between PRAQ scores and child’s sex. Individual PRAQ scores of the second and third trimester and the PRAQ Sum score were analyzed in independent analyses. All analyses included child’s age at MRI scan time (CAM) and child’s sex as (control) variables. In sensitivity analyses, we repeated all the multiple regression analyses by subsequently adding and removing each of the following control variables to/from the model in order to test if the observed results were explained by these covariates: maternal education, BMI, prenatal medication, prenatal alcohol and/or nicotine exposure, pre-/postnatal depression, pre-/postnatal anxiety, gestational complications, previous abortions and/or miscarriages, child’s birth weight and gestational age. In *post hoc* analyses we investigated the association between amygdala volumes and PRAQ scores in semipartial correlation analyses (where we controlled for CAM), in the whole sample and in boys and girls separately.

Finally, we explored the association between PRAQ scores, brain volumes and child behavioral problems (SDQ) in multiple linear regression analyses. Analogous to the method proposed by Baron and Kenny (Baron and Kenny, 1986) for the test of mediation hypotheses, we first investigated the association between SDQ measures (dependent variable) and pregnancy-related anxiety (predictor) with sex as covariate of no interest. Then we investigated the association between SDQ measures and amygdala volumes (predictor) with child’s sex and CAM as covariates of no interest. Then we included both predictors into the same model. Child’s sex and CAM were included as covariates of no interest. We repeated all analyses with sex as an interacting factor to probe sex differences. We also conducted sensitivity analyses as described above, however, considering the sample size, the results of these analyses should be interpreted with caution. We chose a statistical threshold of *p* < 0.05. Given the exploratory nature of the study, no correction for multiple testing was carried out.

## Results

### Description of the Sample – PRAQ Scores and Control Variables

The PRAQ scores of the second and third trimester were highly intercorrelated (*r* = 0.57, *p* = 0.002), and this association was significantly stronger in the mothers of girls (*r* = 0.86) compared to the mothers of boys (*r* = 0.31) (Fisher *z*-transformation; *z* = −2.25, *p* = 0.012). Cross-sectional PRAQ scores did not significantly differ between mothers of girls or boys (Table 1). Mothers of boys reported significantly more gestational complications (Table 1).

**TABLE 1:** The mean scores (*M*) and standard deviations (*SD*) or frequencies, respectively, are listed for maternal prenatal PRAQ scores and control variables, for the whole sample and for girls and boys separately.

**Variable**	**Whole sample**	**Boys (*N* = 14)**	**Girls (*N* = 13)**	***p***
*M* ± *SD (range)*				
Child’s age [mo]	50.8 ± 1.7 (47.7–54.0)	50.8 ± 1.2 (48.7–53.1)	50.8 ± 2.1 (47.7–54.0)	0.948
Gestational weeks	39.9 ± 1.1 (38.0–42.1)	39.9 ± 1.1 (38.0–42.0)	40.0 ± 1.2 (38.0–42.1)	0.854
Birth weight [g]	3627.1 ± 391.6 (2750–4225)	3540.1 ± 431.9 (2750–4025)	3720.8 ± 334.5 (2950–4225)	0.238
PRAQ (gwk 24)	21.85 ± 4.98 (15–36)	22.57 ± 5.33 (17–36)	21.08 ± 4.66 (15–29)	0.447
PRAQ (gwk 34)	21.96 ± 5.96 (13–36)	24.00 ± 5.48 (14–36)	19.77 ± 5.88 (13–30)	0.064
PRAQ Sum	43.81 ± 9.72 (28–61)	46.57 ± 8.73 (32–61)	40.85 ± 10.17 (28–59)	0.128
Prenatal EPDS sum	14.07 ± 9.09 (0–31)	16.36 ± 8.54 (6–31)	11.62 ± 9.35 (0–31)	0.181
Prenatal SCL sum	7.97 ± 8.44 (0–32)	8.30 ± 9.06 (0–32)	7.62 ± 8.08 (0–23)	0.838
Postnatal EPDS sum (*N* = 13)	24.17 ± 20.88 (4–83)	28.03 ± 27.52 (4–83)	19.67 ± 9.64 (8–34)	0.496
Postnatal SCL sum (*N* = 13)	12.62 ± 16.76 (1–60)	16.86 ± 21.36 (2–60)	7.67 ± 8.50 (1–22)	0.346
*Frequencies*				
Maternal pre-pregnancy BMI (< 25/ > = 25)	17/10	7/7	10/3	0.148
Prenatal alcohol and/or nicotine consumption (no/yes)	19/8	9/5	10/3	0.472
Prenatal medication – thyroxine (no/yes)	25/2	13/1	12/1	0.957
Prenatal medication – corticosteroids (no/yes)	25/2	13/1	12/1	0.957
Gestational complications (no/yes)	20/7	8/6	12/1	0.037^*^
Previous miscarriages/abortions (no/yes)	20/7	11/3	9/4	0.580
Maternal education (low/middle/high)	5/5/17	1/3/10	4/2/7	0.287

In the right column *p*-values (of *t*-tests, ANOVAs or chi-squared tests) for sex differences in the sample are listed. ^*^p < 0.05; PRAQ, pregnancy-related anxiety questionnaire; SCL, Symptom Checklist 90; EPDS, Edinburgh Postnatal Depression Scale.

In the whole sample a higher PRAQ score of the third, but not second trimester, and a higher PRAQ Sum score were significantly related to prenatal alcohol and/or nicotine exposure (PRAQ gwk 34: *t* = −2.1, *p* = 0.043; PRAQ Sum: *t* = −2.3, *p* = 0.033) and a higher maternal prenatal EPDS sum score (gwk 34: *r* = 0.49, *p* = 0.010; Sum: *r* = 0.48, *p* = 0.011). PRAQ scores of the second trimester and the PRAQ Sum scores were significantly associated with postnatal depressive symptoms (gwk24: *W* = 3.6, *p* = 0.002; Sum: *W* = 2.8, *p* = 0.012), and PRAQ scores of the second trimester were also significantly related to postnatal anxiety (gwk24: *W* = 3.1, *p* = 0.006). No further significant associations between the PRAQ scores and the control variables were found.

### Description of the Sample – Amygdala Volumes

Girls showed larger bilateral amygdala volumes compared to boys (Table 2). This sex difference in the sample was significant when volumes were corrected for total intracranial volume.

**TABLE 2:** The uncorrected and corrected volumes of left and right amygdala – as assessed by manual segmentation – are listed for the whole sample and for boys and girls separately.

**Volumes [mm^3^] (M ± SD; range)**	**Whole sample**	**Boys (*N* = 14)**	**Girls (*N* = 13)**	***p***
Left amygdala volume	1151.2 ± 104.4 (908–1320)	1115.4 ± 104.9 (908–1273)	1189.7 ± 92.6 (1028–1320)	0.063
Right amygdala volume	1189.8 ± 118.2 (935–1480)	1164.6 ± 127.5 (935–1379)	1217.0 ± 105.3 (1091–1480)	0.257
Left amygdala volume/TIV	0.08 ± 0.01 (0.05–0.11)	0.08 ± 0.01 (0.05–0.09)	0.09 ± 0.01 (0.08–0.11)	<0.001
Right amygdala volume/TIV	0.09 ± 0.01 (0.06–0.12)	0.08 ± 0.01 (0.06–0.09)	0.09 ± 0.01 (0.08–0.12)	<0.001

*In the right column *p*-values of sex differences in the sample are listed. TIV, total intracranial volume.*

### Association Between Amygdala Volumes and Pregnancy-Related Anxiety

#### In the Whole Sample a Larger Left Amygdala Volume Was Significantly Associated With Higher PRAQ Scores of the Third Trimester, but Only After Control for Some Confounders

We did not observe significant associations between amygdala volumes and PRAQ scores in multiple linear regression analyses of the whole sample (controlling for child’s sex and CAM) (Table 3). However, in the sensitivity analyses (including control variables into the model, see Materials and Methods, Statistical Analyses) we found that higher PRAQ scores of the third trimester were significantly associated with larger left relative amygdala volume when we controlled for maternal pre- or postnatal depressive symptoms or postnatal anxiety (*p* < 0.05) (each in separate analyses, see Materials and Methods, Statistical Analyses). The same effects of the control variables were found for the PRAQ Sum score (*p* < 0.05).

**TABLE 3:** The association between amygdala volumes (dependent variable) and PRAQ scores and their interaction with child’s sex was tested in multiple linear regression analyses.

**Volumes**	**PRAQ gwk24**		**PRAQ gwk34**		**PRAQ Sum**	
	**β ± SE**	***p***	**β ± SE**	***p***	**β ± SE**	***p***
Left amy	2.8 ± 4.0	0.497	2.4 ± 3.6	0.512	1.6 ± 2.1	0.450
Left amy/TIV	1.8 ± 3.9 (x10^−4^)	0.647	5.5 ± 3.3 (x10^−4^)	0.108^a^	2.5 ± 2.0 (x10^−4^)	0.231^b^
Right amy	−1.0 ± 4.6	0.826	−3.0 ± 4.1	0.468	−1.4 ± 2.5	0.582
Right amy/TIV	−1.0 ± 4.0 (x10^−4^)	0.809	2.1 ± 3.5 (x10^−4^)	0.562	0.5 ± 2.1 (x10^−4^)	0.828

**Interaction between PRAQ and child’s sex (0 = female, 1 = male) on amygdala volumes**						

	**PRAQ gwk24 × sex**		**PRAQ gwk34 × sex**		**PRAQ Sum × sex**	

Left amy	−16.3 ± 7.8	0.049^c^	−11.5 ± 7.0	0.117	−8.2 ± 4.2	0.062
Left amy/TIV	−22.5 ± 6.8 (x10^−4^)	0.003^d^	−9.3 ± 6.5 (x10^−4^)	0.170	−9.1 ± 3.8 (x10^−4^)	0.026^e^
Right amy	−11.8 ± 9.6	0.230	−10.6 ± 8.2	0.210	−7.6 ± 5.0	0.137
Right amy/TIV	−19.3 ± 7.4 (x10^−4^)	0.015^f^	−8.0 ± 7.1 (x10^−4^)	0.271	−8.5 ± 4.1 (x10^−4^)	0.051^g^

*Significant results (*p* < 0.05) are marked in blue. Amy, amygdala; SE, standard error; gwk, gestational week; TIV, total intracranial volume. ^*a*^*p* < 0.05 controlling for maternal pre- or postnatal depressive symptoms or postnatal anxiety. ^*b*^*p* < 0.05 controlling for maternal pre- or postnatal depressive symptoms or postnatal anxiety. ^*c*^*p* < 0.05 with maternal prenatal anxiety, alcohol and/or nicotine exposure and medication (thyroxine), and *p* > 0.05 with each other control variable. ^*d*^*p* < 0.05 with each control variable. ^*e*^*p* < 0.05 with each control variable except prenatal depressive symptoms (*p* < 0.06), gestational complications (*p* < 0.07), postnatal anxiety (*p* < 0.08), and postnatal depressive symptoms (*p* < 0.14). ^*f*^*p* < 0.05 with each control variable except postnatal anxiety (*p* < 0.11) and gestational complications (*p* < 0.13). ^*g*^*p* < 0.05 controlling for maternal education, alcohol and/or nicotine exposure.*

#### Analyzing Sex Differences, Higher PRAQ Scores of the Second Trimester Were Significantly Associated With Smaller Left Amygdala Volumes in Boys Compared to Girls

**FIGURE 1:**
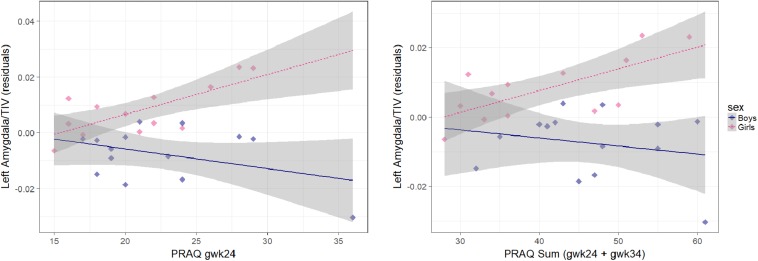
Association between pregnancy-related anxiety (PRAQ) and left corrected amygdala volume [residuals of child’s age (CAM): the influence of CAM has been removed from the variable]. Smaller left amygdala volumes in boys compared to girls were significantly associated with higher PRAQ scores of the second trimester [β = –22.5 ± 6.8 (x10^−4^), *p* = 0.003] and with higher PRAQ Sum scores [β = –9.1 ± 3.8 (x10^−4^), *p* = 0.026].

In a second step, we investigated in multiple linear regression analyses whether the association between amygdala volumes and the individual PRAQ scores significantly differs between girls and boys (controlling for CAM) (Table 3). The PRAQ scores of each trimester and the Sum score were analyzed in separate models. We observed significant sex differences in the association of pregnancy-related anxiety of the second trimester with bilateral relative and left uncorrected amygdala volumes (Table 3 and Figure 1). PRAQ Sum scores showed similar, but weaker sex differences in their association with amygdala volumes. In more detail, higher PRAQ scores were associated with smaller amygdala volumes in boys compared to girls.

In sensitivity analyses, testing potential confounders, the interaction effect of sex and pregnancy-related anxiety of the second trimester remained significant for left relative amygdala volume (*p* < 0.05). The interaction effect of the PRAQ Sum score and sex on right relative amygdala volume became significant when controlling for maternal education, alcohol and/or nicotine exposure. However, the interaction effects of pregnancy-related anxiety and sex were partly reduced to insignificance for left uncorrected and right relative amygdala volumes (PRAQ gwk24) as well as for left relative amygdala volume (PRAQ Sum) (e.g., by gestational complications and maternal postnatal depressive symptoms and anxiety) (Table 3).

#### In *post hoc* Analyses, Higher PRAQ Scores of the Second and Third Trimester Significantly Correlated With Larger Left Amygdala Volume in Girls

In semi-partial correlational analyses of the whole sample we did not observe significant associations between PRAQ scores and amygdala volumes. Analyzing boys and girls separately, we found significant positive correlations between pregnancy-related anxiety in the second and third trimester and left amygdala volumes in girls, while in boys pregnancy-related anxiety in the second trimester was (insignificantly) negatively associated with amygdala volumes (Table 4). Results of the zero-order correlational analyses were similar.

**TABLE 4:** The association between amygdala volumes and PRAQ scores of the second and third trimester and their sum scores is shown.

**Volumes**	**Whole sample**			**Boys (*N* = 14)**			**Girls (*N* = 13)**		
	**PRAQ gwk24**	**PRAQ gwk34**	**PRAQ sum**	**PRAQ gwk24**	**PRAQ gwk34**	**PRAQ sum**	**PRAQ gwk24**	**PRAQ gwk34**	**PRAQ sum**
Left amy	0.07 (−0.32/0.44)	−0.02 (−0.39/0.37)	0.03 (−0.36/0.40)	−0.19 (−0.66/0.38)	−0.18 (−0.65/0.39)	−0.23 (−0.68/0.34)	0.60^*^ (0.07/0.86)	0.49 (−0.09/0.82)	0.56^*^ (0.01/0.85)
Left amy/TIV	−0.03 (−0.40/0.36)	−0.01 (−0.39/0.38)	−0.02 (−0.40/0.36)	−0.39 (−0.76/0.18)	0.04 (−0.50/0.56)	−0.21 (−0.67/0.36)	0.73^∗∗^ (0.30/0.91)	0.63^*^ (0.13/0.88)	0.70^∗∗^ (0.24/0.90)
Right amy	−0.08 (−0.45/0.31)	−0.23 (−0.56/0.17)	−0.18 (−0.52/0.22)	−0.24 (−0.68/0.33)	−0.35 (−0.74/0.22)	−0.37 (−0.75/0.20)	0.28 (−0.32/0.72)	0.12 (−0.46/0.63)	0.20 (−0.40/0.67)
Right amy/TIV	−0.14 (−0.49/0.26)	−0.14 (−0.49/0.26)	−0.15 (−0.50/0.24)	−0.33	−0.14	−0.32	0.52 (−0.05/0.83)	0.39 (−0.21/0.77)	0.46 (−0.12/0.81)

The Pearson Product Moment correlation coefficients (with the 95% confidence intervals in parentheses) or Spearman correlation coefficients of the semipartial correlations (controlling for CAM) are listed for the whole sample and for boys and girls separately (significant results are marked in blue). amy, amygdala; ^*^p < 0.05, ^∗∗^*p* < 0.01.

### In Whole-Brain Analyses, No Significant Associations Between PRAQ Scores and Brain Gray Matter Volumes Were Observed

In the whole-brain analyses, no significant associations between pregnancy-related anxiety (both time points and sum score) and brain gray matter volume were observed. Analyzing boys and girls separately, no significant associations were found either. Applying a more lenient statistical threshold (see Material and Methods, Whole Brain Analysis), we observed in the whole sample that higher PRAQ gwk24 scores were related to smaller left temporal volumes, and higher PRAQ gwk34 were related to greater left precuneus volumes (Supplementary Table SI-1). Comparing boys and girls, bilateral middle cingulate and right superior parietal and occipital volumes were more positively correlated with PRAQ gwk24, and left cerebellar and parahippocampal gyrus volumes were more negatively associated with PRAQ gwk34 in boys compared to girls (Supplementary Table SI-1).

### Association of Pregnancy-Related Anxiety and Brain Volumes With Behavioral Problems at the Child Age of 4 Years

#### In the Whole Sample, Higher PRAQ Scores of the Second Trimester Were Related to More Emotional Symptoms and Higher SDQ Sum Scores

First, we explored in multiple regression analyses whether pregnancy-related anxiety is associated with child behavioral problems reported at 4 years. Descriptive information of the SDQ measures is presented in Table 5.

**TABLE 5:** Descriptive information for the SDQ measures of the 4-year-olds.

	**Four-Year-Old children**			
**Variable (M ± SD; range)**	**Whole sample (*N* = 21)**	**Boys (*N* = 12)**	**Girls (*N* = 9)**	***p***
SDQ-F1	1.29 ± 1.35 (0–6)	1.33 ± 1.72 (0–6)	1.22 ± 0.67 (0–2)	0.857
SDQ-F2	3.57 ± 1.69 (0–7)	4.17 ± 1.40 (2–7)	2.78 ± 1.79 (0–5)	0.060
SDQ-F3	3.29 ± 2.19 (0–7)	4.08 ± 2.15 (1–7)	2.22 ± 1.86 (0–5)	0.052
SDQ-F4	2.05 ± 2.09 (0–8)	2.25 ± 2.49 (0–8)	1.78 ± 1.48 (0–4)	0.620
SDQ Sum	10.19 ± 5.25 (1–25)	11.83 ± 5.77 (4–25)	8.00 ± 3.71 (1–15)	0.099

*SDQ-F1, emotional symptoms; SDQ-F2, conduct problems; SDQ-F3, hyperactivity/inattention; SDQ-F4, peer relationship problems.*

We found that higher pregnancy-related anxiety in the second trimester is significantly associated with more emotional symptoms (β = 0.13 ± 0.06, *p* = 0.035) and a higher SDQ Sum score (β = 0.43 ± 0.20, *p* = 0.048) in the whole sample. The association between PRAQ gwk24 scores and emotional symptoms was reduced to insignificance by control for maternal postnatal depressive symptoms and anxiety (*p* > 0.5). The associations with SDQ Sum scores were insignificant with most control variables.

#### Analyzing Sex Differences, Higher PRAQ Scores Were Significantly Associated With More Emotional Symptoms in Boys Compared to Girls

Furthermore, we detected significant sex differences: A higher pregnancy-related anxiety in the second trimester (β_boys_ = 0.26 ± 0.11, *p* = 0.036) and a higher PRAQ Sum score (β_boys_ = 0.14 ± 0.06, *p* = 0.027) were significantly related to more emotional symptoms in boys compared to girls. The sex-specific association of PRAQ gwk24 with emotional symptoms remained significant even after controlling for each confounder, but was reduced to insignificance for PRAQ Sum by maternal prenatal (*p* = 0.067) and postnatal depressive symptoms (*p* = 0.087).

#### Larger Bilateral Amygdala Volumes Were Associated With Partly Lower SDQ Scores

We further explored whether amygdala volumes that were associated with pregnancy-related anxiety might also be related to child behavioral problems. We found that a larger left relative amygdala volume was significantly associated with less emotional symptoms (β = −65.0 ± 28.2, *p* = 0.033). A larger right relative amygdala volume was significantly related to less peer relationship problems (β = −113.0 ± 43.3, *p* = 0.017). No further significant effects were found. In the sensitivity analyses, associations were reduced to insignificance by maternal pre- and postnatal depressive symptoms, postnatal anxiety and for right relative amygdala volume additionally by birth weight.

#### Analyzing Sex Differences, a Larger Left Amygdala Volume Was Associated With Less Emotional Symptoms, and Larger Bilateral Amygdala Volumes With Less Peer Relationship Problems in Boys Compared to Girls, but Only After Inclusion of Some Confounders

Furthermore, significant sex differences emerged by inclusion of some of the control variables (such as medication) into the models: In boys compared to girls, emotional symptoms were significantly more negatively associated with left relative amygdala volume, and peer relationship problems were significantly more negatively related to left relative and uncorrected and right relative amygdala volumes.

#### Amygdala Volumes Did Not Mediate the Association Between PRAQ and SDQ Scores in the Whole Sample, but Might Partly Mediate Observed Sex Differences

**FIGURE 2:**
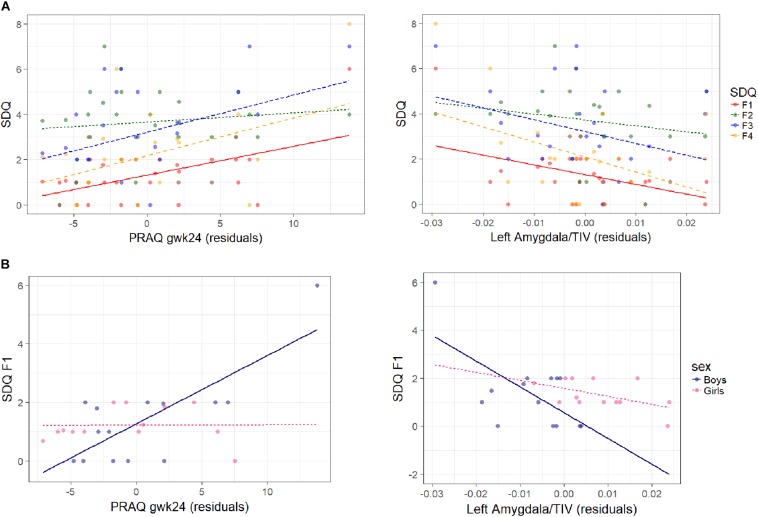
Association between SDQ measures, PRAQ gwk24 scores and left corrected amygdala volumes are shown **(A)** for all SDQ measures in the whole sample, and **(B)** for emotional symptoms in boys and girls separately. On the left the association of SDQ measures with residuals of PRAQ gwk24 is displayed [residuals: the influence of left relative amygdala volume and child’s age (CAM) has been removed from the variable]. On the right the association of SDQ measures with residuals of left relative amygdala volume is shown [residuals: the influence of PRAQ gwk24 and child’s age (CAM) has been removed from the variable] (F1: emotional symptoms, F2: conduct problems, F3: hyperactivity/inattention, F4: peer relationship problems). Higher PRAQ gwk24 scores were significantly associated with more emotional symptoms (β = 0.13 ± 0.06, *p* = 0.035) (and with a higher SDQ Sum score, not shown) in the whole sample **(A left)**, and with more emotional symptoms in boys compared to girls (β_boys_ = 0.26 ± 0.11, *p* = 0.036) **(B left)**. A larger left amygdala volume was significantly associated with less emotional symptoms (β = –65.0 ± 28.2, *p* = 0.033) in the whole sample **(A right)**, and with less emotional symptoms in boys compared to girls (significant in some control analyses) **(B right)**.

In order to test whether amygdala volumes mediate the association between PRAQ scores and SDQ, we included both pregnancy-related anxiety and amygdala volume as predictors into the regression models. Results are presented in Table 6 and Figure 2. We found that higher pregnancy-related anxiety in the second trimester was still significantly associated with more emotional symptoms and a higher SDQ Sum score. Furthermore, we additionally observed a significant association of the PRAQ Sum score with emotional symptoms and peer relationship problems when left relative amygdala volume was included into the model. Greater left and right relative amygdala volumes were still significantly associated with fewer emotional symptoms and less peer relationship problems, respectively. Additionally, a larger left relative amygdala volume was also related to less peer relationship problems and a lower SDQ Sum score. Associations were partly reduced to insignificance by control variables, but remained significant for the association between left (relative and uncorrected) amygdala volume and emotional symptoms with all control variables except postnatal anxiety (left relative amygdala volume: *p* = 0.066; left uncorrected amygdala volume: *p* = 0.102). The sexually dimorphic associations of (a) pregnancy-related anxiety with emotional symptoms, and (b) amygdala volumes with emotional symptoms and peer relationship problems were all statistically insignificant when including both predictors into the models. The sensitivity analyses corroborated these findings, exempt that significant sex differences were observed with some of the control variables for the association between pregnancy-related anxiety and emotional symptoms in models that included right relative or left uncorrected amygdala volumes, but not left relative amygdala volume.

**TABLE 6:** Association between pregnancy-related anxiety, amygdala volume and child behavioral problems.

	**SDQ-F1**	**SDQ-F2**	**SDQ-F3**	**SDQ-F4**	**SDQ SUM**
*Model 1:* PRAQ gwk24 AmyL	β = 0.15 ± 0.05, *p* = 0.012 β = −0.01 ± 0.00, *p* = 0.030	β = 0.04 ± 0.08, *p* = 0.607 β = 0.01 ± 0.00, *p* = 0.240	β = 0.11 ± 0.10, *p* = 0.264 β < −0.01 ± 0.01, *p* = 0.596	β = 0.17 ± 0.09, *p* = 0.084 β = -0.01 ± 0.01, *p* = 0.103	β = 0.47 ± 0.20, *p* = 0.030 β = −0.01 ± 0.01, *p* = 0.230
*Model 2:* PRAQ gwk24 AmyL/TIV	β = 0.16 ± 0.05, *p* = 0.003 β = −73.4 ± 22.5, *p* = 0.005	β = 0.05 ± 0.08, *p* = 0.552 β = 10.8 ± 42.8, *p* = 0.804	β = 0.11 ± 0.11, *p* = 0.303 β = −47.8 ± 52.9, *p* = 0.379	β = 0.17 ± 0.09, *p* = 0.072 β = −105.2 ± 45.3, *p* = 0.034	β = 0.48 ± 0.18, *p* = 0.016 β = -215.7 ± 98.1, *p* = 0.042
*Model 3:* PRAQ gwk24 AmyR/TIV	β = 0.14 ± 0.05, *p* = 0.011 β = −50.1 ± 24.1, *p* = 0.052	β = 0.05 ± 0.08, *p* = 0.512 β = 31.3 ± 38.1, *p* = 0.423	β = 0.10 ± 0.11, *p* = 0.371 β = −51.7 ± 47.9, *p* = 0.295	β = 0.14 ± 0.09, *p* = 0.128 β = −110.7 ± 42.3, *p* = 0.018	β = 0.43 ± 0.19, *p* = 0.036 β = −181.3 ± 92.5, *p* = 0.065
*Model 4:* PRAQ Sum AmyL/TIV	β = 0.07 ± 0.03, *p* = 0.021 β = −87.5 ± 28.4, *p* = 0.007	β = 0.01 ± 0.05, *p* = 0.918 β = 11.7 ± 41.8, *p* = 0.783	β = 0.04 ± 0.05, *p* = 0.471 β = −48.5 ± 53.5, *p* = 0.377	β = 0.11 ± 0.04, *p* = 0.024 β = −112.0 ± 43.3, *p* = 0.019	β = 0.22 ± 0.11, *p* = 0.056 β = −236.3 ± 104.1, *p* = 0.036
*Model 5:* PRAQ Sum AmyR/TIV	β = 0.05 ± 0.03, *p* = 0.109 β = −58.8 ± 28.5, *p* = 0.055	β = 0.01 ± 0.05, *p* = 0.890 β = 28.1 ± 38.8, *p* = 0.479	β = 0.03 ± 0.05, *p* = 0.582 β = −50.8 ± 47.1, *p* = 0.295	β = 0.09 ± 0.04, *p* = 0.054 β = −118.4 ± 40.5, *p* = 0.010	β = 0.17 ± 0.11, *p* = 0.131 β = −199.8 ± 99.7, *p* = 0.060

*AmyL, left amygdala volume; AmyR, right amygdala volume; gwk, gestational week; SDQ-F1, emotional symptoms; SDQ-F2, conduct problems; SDQ-F3, hyperactivity/inattention; SDQ-F4, peer relationship problems.*

## Discussion

With this study we investigated whether maternal pregnancy-related anxiety – a measure of maternal prenatal distress – is associated with brain gray matter volume differences in 4-year-old children, and whether the observed brain structural changes mediate the association between maternal pregnancy-related anxiety and behavioral/emotional difficulties in children.

We found that maternal pregnancy-related anxiety in the second, but not third trimester was significantly more positively associated with bilateral relative amygdala volumes in girls compared to boys. The sexually dimorphic association between pregnancy-related anxiety and left relative amygdala volume remained significant after controlling for pre- and postnatal confounders. In addition, higher maternal pregnancy-related anxiety in the third trimester was significantly positively associated with larger left amygdala volume in the whole sample after controlling for confounders, but this positive association was primarily driven by the female subsample. The PRAQ Sum score – a proxy for the chronicity of stress exposure – showed a very similar, but slightly weaker pattern, and did not reveal any additional effects. In summary, our results support our hypothesis that maternal pregnancy-related anxiety is associated with sexually dimorphic alterations in amygdala volume, independent of other pre- and postnatal factors. However, in our whole-brain analyses, we observed only weak, statistically insignificant associations of pregnancy-related anxiety with (sub)cortical brain areas including sexually dimorphic associations with the parahippocampal gyrus, and cingulate, parietal, occipital and cerebellar cortices.

Pregnancy-related anxiety and bilateral amygdala volumes were both linked to child emotional/behavioral difficulties (i.e., emotional symptoms, peer relationship problems, SDQ Sum score): Higher pregnancy-related anxiety in the second trimester and a higher PRAQ Sum score were associated with more behavioral difficulties, and these associations remained significant after controlling for amygdala volumes, but were reduced to insignificance by some confounders. The association of pregnancy-related anxiety with emotional symptoms was partly sexually dimorphic, and while this sex-specific interaction effect survived statistical control for all confounders, it was reduced to insignificance by inclusion of left relative amygdala volume into the model. On the contrary, larger amygdala volumes were related to less behavioral difficulties. The association between left amygdala volume and emotional symptoms remained significant even after controlling for pregnancy-related anxiety and confounders except postnatal anxiety.

In our sample, pregnancy-related anxiety was moderately correlated between the two time points as has been reported by Huizink et al. (2016). Furthermore, we observed higher amygdala volumes in girls compared to boys. So far, sex differences of amygdala brain volumes have been inconsistent in children (Sowell et al., 2002; Lenroot and Giedd, 2010; Buss et al., 2012), but in adults amygdala volumes were consistently larger in males compared to females presumably as a consequence of androgen-related maturational growth during puberty (Giedd et al., 1997; Merke et al., 2003; Ruigrok et al., 2014).

### Prenatal Stress and Amygdala Volume

Structural alterations in limbic areas following prenatal stress have frequently been observed in animal studies (Bock et al., 2015). In children, a larger right amygdala volume in girls, but not boys, was related to higher maternal salivary cortisol and to higher maternal depressive symptoms in the second trimester of gestation (assessed at gwk 15 and 26, respectively) (Buss et al., 2012; Wen et al., 2017). Therefore, our results provide further support that maternal prenatal stress in the second trimester is linked to sexually dimorphic structural alterations in the amygdala. Our results also showed for the first time that pregnancy-related anxiety in the third trimester is related to larger left amygdala volumes (driven by the female subsample). The amygdala starts to develop early in embryonic life: Around the fifth week of gestation all three main subdivisions of the amygdaloid complex can be identified (Humphrey, 1968; Müller and O’Rahilly, 2006) and after the eighth week important connections to other components of the limbic system are established (Müller and O’Rahilly, 2006). During the second trimester, migration of immature amygdaloid neurons, neuronal differentiation and axonal outgrowth take place. Intense synaptogenesis is observed until the early third trimester, and in the eighth month the amygdala reaches an adult-like anatomical shape (Ulfig et al., 2003). It has been proposed that the brain is particularly vulnerable when its neural circuits are in the process of becoming organized (Graignic-Philippe et al., 2014). It has further been suggested that neurodevelopmental trajectories differ between sexes and that this might lead to sex-specific time windows of vulnerability (Buss et al., 2009; Bock et al., 2015; Entringer et al., 2015). A sexually dimorphic developmental trajectory of the amygdala might explain why prenatal stress is associated with sex differences in the second, but not in the third trimester.

Higher pregnancy-related anxiety has been shown to be associated with higher fetal cortisol exposure (Dahlerup et al., 2018), and glucocorticoids might convey the effects of pregnancy-related anxiety on amygdala volumes during gestation. Neuronal differentiation and axon guidance are inhibited by glucocorticoids, while glial cell proliferation is promoted (Moors et al., 2012). Increases in glial and neuronal cells in the lateral part of the amygdala due to prenatal stress were found in adult male rats and resulted in a larger amygdala volume (Salm et al., 2004). However, in human neonates, maternal prenatal depression was not associated with alterations in amygdala volumes (Rifkin-Graboi et al., 2013), and the association between pregnancy-related anxiety and neonatal amygdala volume has not been studied so far. It is conceivable that the effects of prenatal stress on amygdala volume evolve postnatally, either by altered postnatal developmental trajectories of the amygdala or by altered child’s stress reactivity, or both. Supporting this notion, amygdalar nuclei were shown to develop differently after birth in young male rats that had been exposed to prenatal stress (Kraszpulski et al., 2006), and they exhibited higher corticotropin releasing hormone (CRH) levels and more CRH receptors in the amygdala (Weinstock, 2007). Prenatal stress was associated with a dysregulation of the HPA axis in animal offspring, and prenatally stressed rats showed chronic anxiety and a lack of adaptation to environmental stressors (Weinstock, 2007). In humans, the majority of studies has reported that higher prenatal maternal distress is associated with greater negative reactivity and impaired self-regulation in children (Korja et al., 2017). Furthermore, pregnancy-related anxiety has been found to modulate infant’s cortisol response to stressors (Tollenaar et al., 2011). A higher stress reactivity renders the individual more susceptible to environmental stressors, which might result in more chronic stress experiences and could subsequently affect amygdala volumes. In adult male rats, acute and chronic stress caused a hypertrophy of the basolateral amygdala: Higher spine density was observed after acute and chronic immobilization stress (Vyas et al., 2002; Mitra et al., 2005), and repeated social defeat and chronic immobilization stress increased dendritic arborization (Patel et al., 2018). The hypertrophy of amygdala persisted even after a stress-free recovery period (Vyas et al., 2004). In humans, a larger amygdala volume was also associated with a longer period of early adverse rearing conditions (i.e., institutional rearing) (Tottenham et al., 2010). Noradrenergic functions that are activated by emotional arousal and are indispensable for fear memory consolidation might be involved in this stress-induced growth of the amygdala (Roozendaal et al., 2009).

Conversely, a smaller amygdala volume, especially in the left hemisphere, has been reported in children with congenital adrenal hyperplasia who are characterized by low cortisol and high androgen levels prenatally, but partly excess cortisol levels postnatally due to medical treatment (Merke et al., 2003; Rose et al., 2004). Smaller amygdala volumes, predominantly in the left hemisphere, were also observed in adults receiving chronic corticosteroid therapy (Brown et al., 2008) and in hypercortisolic children diagnosed with Cushing’s syndrome, and these alterations in amygdalar volume persisted after correction for hypercortisolism (Merke et al., 2005). In sum, while higher stress exposure might be related to larger amygdala volumes, higher cortisol exposure without emotional arousal (as in the above-mentioned medical conditions) or an excess of cortisol might lead to smaller amygdala volumes, especially in the left hemisphere.

In our study, pregnancy-related anxiety in the second trimester was associated with larger amygdala volumes in girls compared to boys. Sexually dimorphic outcomes of maternal prenatal distress have been reported in several studies, but the mechanisms are not yet clear (Buss et al., 2012; Braithwaite et al., 2017, 2018; Wen et al., 2017; Soe et al., 2018). Gonadal hormones modulate amygdalar responses to anxiogenic stimuli (Gray and Bingaman, 1996) and sexually dimorphic alterations in brain neurotransmitter systems in cortical areas (e.g., turnover rates of dopamine and serotonin) were observed in rats following prenatal stress (Weinstock, 2007). It has been suggested that the fetal placenta might be partly responsible for the observed sex differences. Sexually dimorphic placental functions have been reported in several studies: for instance, a sex-specific distribution of placental glucocorticoid receptor subtypes has been observed (St-Pierre et al., 2016), and the placental epigenetic machinery regulating gene expression showed pronounced sexually dimorphic responses to prenatal stress (Bock et al., 2015; St-Pierre et al., 2016), such as sex-specific placental expression of glucocorticoid receptors, of genes implicated in immune pathways, of insuline-like growth factors and of 11β-HSD2, the placental barrier for cortisol (Clifton, 2010). It was found that the female placenta adjusts gene expression to heightened maternal cortisol levels by upregulation of 11β-HSD2 and by downregulation of glucocorticoid receptors while no comparable adaptations were seen in male placentas. Postnatally increased cortisol concentrations were found in female neonates compared to males after exposure to elevated prenatal cortisol. It has been suggested that the adjustments in the female placenta preserve female fetal adrenal functions and might be involved in the higher survival rate of female compared to male fetuses who lack this adaptive response in situations of high maternal prenatal distress (Clifton, 2010). Animal and human studies nevertheless also showed that females displayed elevated fear reactions and a more negative emotionality, indicating a higher stress reactivity, after increased prenatal cortisol exposure, while no or opposite effects were seen in males (Weinstock, 2007; Clifton, 2010; Braithwaite et al., 2017).

In summary, heightened maternal cortisol was associated with more physiological adaptations in female compared to male placentas which might preserve the adrenal functions of the female offspring, but were also associated with elevated stress reactivity in females postnatally. We assume that the more positive association between amygdala volume and pregnancy-related anxiety in girls compared to boys arises from a higher postnatal stress reactivity in girls and a subsequently evolving hypertrophy of the amygdala. The partly negative association between pregnancy-related anxiety and amygdala volume in boys rather resembles the effects that emerge after exposure to elevated glucocorticoids without emotional arousal (Merke et al., 2003, 2005; Rose et al., 2004; Brown et al., 2008). Most of these studies revealed that a hypercortisolic state is accompanied by amygdalar volume reductions predominantly in the left hemisphere (Merke et al., 2003; Rose et al., 2004; Brown et al., 2008). We speculate that higher pregnancy-related anxiety evokes a hypercortisolic state in males, presumably via changing adrenal functioning pre- and/or postnatally, that causes a smaller amygdala volume, predominantly in the left hemisphere.

### Amygdala and Child Behavior

While other studies have reported associations between prenatal distress and the right amygdala volume, in our study, associations between pregnancy-related anxiety and amygdala were more pronounced for the left than the right hemisphere. While both left and right amygdala seem to be equally implicated in the evaluation and encoding of emotional stimuli, in arousal and attention, there is some evidence that the left amygdala might be more strongly involved in fear and in more explicit emotion processing, while the right amygdala might contribute more to implicit, global emotion processing and emotional memory retrieval (Markowitsch, 1999). An early lesion in the left amygdala was associated with a severely impaired ability to represent mental states (“Theory of mind”), an important social skill (Fine et al., 2001). In addition, several studies have revealed a smaller left amygdala volume in children with conduct problems (Rogers and De Brito, 2016). Smaller amygdala volumes were also linked to more proactive aggression (Naaijen et al., 2018) and a diagnosis of schizophrenia (Fischer et al., 2012). On the other hand, a larger left amygdala volume significantly predicted more anxiety in 7–9 years old children (Qin et al., 2014), but was also associated with better cognitive mental state inference in 4-year-olds (Rice et al., 2014).

Interestingly, in our sample, a larger amygdala volume – especially of the left hemisphere – was related to less emotional symptoms, less peer relationship problems and less overall child difficulties (SDQ Sum score). This effect might be explained by enhanced aggression and reduced theory-of-mind skills in association with smaller left amygdala volumes. Even though we would have expected more emotional problems in girls with a larger amygdala volume (considering the positive association between amygdala volumes and anxiety) the sensitivity of the SDQ parent report for child anxiety and depression is not very high and might well account for this lack of association (Goodman et al., 2000).

Left relative amygdala volumes might partly mediate the sexually dimorphic association between pregnancy-related anxiety and emotional symptoms because its inclusion into the statistical model weakened the interaction effects. But beyond that, our data offered little support for the notion that the association between pregnancy-related anxiety and child difficulties is mediated by amygdala volumes. By contrast, our data suggest that both pregnancy-related anxiety and amygdala volumes are independent predictors of child difficulties. Hence, pregnancy-related anxiety seems to affect child behavior in parts via mechanisms that we could not detect with our brain structural study and might involve changes in connectivity, distribution of neurotransmitters and receptors among others. Furthermore, we cannot rule out that genetic factors are involved in the association between pregnancy-related anxiety and behavioral problems. For instance, a higher maternal anxiety-proneness could be partly genetically determined and genetically transmitted to the next generation leading to less self-regulation capacities in the child.

In our whole-brain analyses we found only weak associations between pregnancy-related anxiety and (sub)cortical gray matter volumes. Interestingly, we observed (weak) sexually dimorphic gray matter alterations in brain areas that contain a high density of steroid receptors such as cingulate, and parietal cortices, insulae and parahippocampal regions (Goldstein et al., 2001; Lenroot and Giedd, 2010). One earlier study has reported gray matter reductions in several cortical areas in association with higher pregnancy-related anxiety in the early second trimester (gwk 19), but not at later time points in gestation (gwk 25 and 31) (Buss et al., 2010). It is therefore possible that we could not detect significant gray matter alterations because of the time point of assessment (gwk 24) in our study.

### Limitations

Several limitations of our study have to be mentioned. The sample size of our study was rather small and data for child difficulties was partly incomplete which might reduce power and limit the generalizability of our study results. A replication of the study results is warranted.

Child difficulties were assessed by maternal report and might be biased, e.g., by maternal postnatal depression or anxiety, even though we controlled for possible postnatal confounders. It would be worthwhile if future studies would include more objective behavioral measures for the assessment of child difficulties, or if future studies would investigate the child’s stress reactivity, e.g., by assessing the cortisol response to experimental stressors, or the child’s chronic stress level, e.g., by measuring hair cortisol levels.

Pregnancy-related anxiety was assessed twice (gwk 24 and gwk 34), but results of other studies suggest that an additional assessment time point earlier during gestation would be valuable, too. As aforementioned, pregnancy-related anxiety was shown to be associated with a downregulation of the placental barrier for cortisol (11β-HSD2) (Dahlerup et al., 2018), but we have not measured the enzymatic activity of 11β-HSD2 at the end of pregnancy in our sample which would add an objective measure to the self-reported PRAQ scores.

The quality of the postnatal environment can modify prenatal risk conditions (Entringer et al., 2015). While we controlled for maternal postnatal anxiety and depressive symptoms, it was beyond the scope of this study to take other postnatal environmental factors into account such as the early caregiving behavior. Early caregiving behavior plays an important role for the child’s socioemotional and cognitive development, as well as for the development of brain circuits (e.g., Harlow and Harlow, 1962; Sheridan et al., 2012; Tyrka et al., 2013). For instance, child abuse and childhood attachment insecurity have been associated with alterations of brain volumes and brain activity in child- and/or adulthood (e.g., Hane and Fox, 2006; Hart and Rubia, 2012; Tyrka et al., 2013; Schneider-Hassloff et al., 2016; Teicher et al., 2016). We suggest that future studies further address the interplay between prenatal risk factors and postnatal environmental quality on child’s brain development. It was also beyond the scope of this study to unravel the effects of maternal traumatic experiences (e.g., during pregnancy) on child’s brain development, and we propose that future studies target this highly important topic.

Finally, recent studies provide evidence that genetic factors interact with prenatal stress on infant brain volumes (Qiu et al., 2015, 2017), and the investigation of gene-environment interactions seems to be a promising avenue for the next steps in research on human development.

## Conclusion

Our results provide further support for the theory of “developmental origins of health and disease.” Pregnancy-related anxiety in the second trimester was associated with larger left amygdala volumes in girls compared to boys, and both pregnancy-related anxiety and amygdala volumes were associated with emotional and behavioral difficulties at 4 years of age.

## Data Availability

The datasets for this study will not be made publicly available because of Finnish data protection legislation. Requests to access the datasets should be directed to HK (hasseka@utu.fi).

## Author Contributions

HA performed the data analyses (preprocessing, manual segmentation of the amygdala, VBM and statistical analyses), interpreted the data, and drafted the manuscript. NH performed the manual segmentation. JP supported the statistical analyses. VS, RP, and TL provided the technical and clinical support for the MRI data acquisition. VS provided the description of the MRI data acquisition methods. JT and NS were involved in the planning and funding of the study. JT, OR, and TIL collected the MRI data. HK planned and established the Cohort and provided funding and infrastructure for the collection of the questionnaire data and the brain imaging and took part in the drafting of the manuscript. LK co-planned and established the Cohort with HK and participated in providing funding for the data collection. All co-authors revised the manuscript and accepted the final manuscript version.

## Conflict of Interest Statement

The authors declare that the research was conducted in the absence of any commercial or financial relationships that could be construed as a potential conflict of interest.

## Footnotes

1 http://www.finnbrain.fi

2 http://www.thl.fi

3 http://www.itksnap.org

4 http://www.bic.mni.mcgill.ca/software/Display/Display.html

5 http://www.fil.ion.ucl.ac.uk/spm/

6 http://www.fz-juelich.de/inm/inm-1/DE/Forschung/_docs/SPMAnatomyToolbox/SPMAnatomyToolbox_node.html

7 fmri.wfubmc.edu

8 http://www.r-project.org/
