# Supplementary material for: Maternal Pregnancy-Related Anxiety Is Associated With Sexually Dimorphic Alterations in Amygdala Volume in 4-Year-Old Children
Source: Front Behav Neurosci. 2019 Aug 6;13:175. doi: 10.3389/fnbeh.2019.00175 (PMC6691065; doi:10.3389/fnbeh.2019.00175)
Supplement: Supplementary file 1 [file Table_1.doc]

**Supplementary Information**

**Table SI-1:**

The results of the whole-brain analyses for the association between pregnancy-related anxiety and brain gray matter volume are shown. Only contrasts with significant results (p < 0.001 uncorrected, kmin = 50 voxels, t ≥ 5.0) are listed.

| **Anatomical region** | **Brain Area** |  | **x** | **y** | **z** | **t** | **k** | **p(FWE, peak)** |
| --- | --- | --- | --- | --- | --- | --- | --- | --- |
| PRAQ gwk24 | | | | | | | | |
| *Whole sample - negative correlation* | | | | | | | | |
| Middle and superior temporal gyrus |  | L | -51 | -30 | -9 | 5.26 | 254 | 0.336 |
| *Boys – positive correlation* | | | | | | | | |
| Inferior frontal gyrus (pars triangularis), middle frontal gyrus | 45 | L | -42 | 26 | 28 | 6.06 | 416 | 0.091 |
| *Boys > girls* | | | | | | | | |
| Middle occipital gyrus |  | R | 38 | -76 | 22 | 5.60 | 66 | 0.197 |
| Middle cingulate cortex | 5Ci (SPL) | R | 8 | -19 | 39 | 5.51 | 453 | 0.228 |
| Superior parietal lobule and occipital gyrus, angular gyrus | 7A, 7P (SPL) | R | 23 | -63 | 46 | 5.49 | 147 | 0.234 |
| Middle cingulate cortex, precuneus | 5Ci/5M (SPL) | L | -15 | -36 | 37 | 5.04 | 250 | 0.456 |
| PRAQ gwk34 | | | | | | | | |
| *Whole sample - positive correlation* | | | | | | | | |
| Precuneus, paracentral lobule | 5M (SPL), 3a, 4a, 5L | L | -11 | -45 | 61 | 5.54 | 94 | 0.219 |
| *Boys – positive correlation* | | | | | | | | |
| Precuneus | 5M (SPL), 3a, 4a | L | -9 | -45 | 61 | 5.62 | 127 | 0.192 |
| Post- and precentral gyrus, supramarginal gyrus | 2,3b,1,PFt | R | 51 | -22 | 43 | 5.07 | 566 | 0.441 |
| *Boys – negative correlation* | | | | | | | | |
| Insula, superior temporal gyrus, Heschl’s gyrus | Ig2, Id1, TE 1.0, OP4 | R | 42 | -12 | -8 | 5.08 | 205 | 0.439 |
| *Girls – positive correlation* | | | | | | | | |
| Parahippocampal gyrus, amygdala, superior temporal pole, entorhinal cortex | LB, Fo3, SF | R | 14 | 2 | -21 | 5.61 | 311 | 0.197 |
| Cerebellar hemisphere, fusiform gyrus, parahippocampal gyrus, subiculum | Lobule I-V | L | -14 | -36 | -15 | 5.33 | 174 | 0.306 |
| *Girls – negative correlation* | | | | | | | | |
| Middle cingulate cortex, supplementary motor area |  | L/R | 2 | 8 | 45 | 5.19 | 151 | 0.372 |
| *Boys < girls* | | | | | | | | |
| Cerebellar hemisphere, parahippocampal gyrus, fusiform gyrus, lingual gyrus, subiculum | Lobule I-V | L | -14 | -37 | -14 | 5.88 | 312 | 0.124 |
| PRAQ Sum | | | | | | | | |
| *Boys – positive correlation* | | | | | | | | |
| Inferior frontal gyrus (pars triangularis), middle frontal gyrus | 45 | L | -39 | 26 | 28 | 5.48 | 159 | 0.243 |
| Postcentral gyrus | 2, 3b, PFt, 1 | R | 50 | -24 | 45 | 5.40 | 451 | 0.274 |
| *Girls – positive correlation* | | | | | | | | |
| Parahippocampal gyrus, amygdala, entorhinal cortex | LB, SF | R | 14 | 2 | -21 | 5.27 | 187 | 0.333 |
| *Girls – negative correlation* | | | | | | | | |
| Middle cingulate cortex, supplementary motor area |  | L/R | 2 | 8 | 45 | 5.09 | 205 | 0.428 |
| *Boys > girls* | | | | | | | | |
| Cerebellar hemisphere, lingual gyrus, fusiform gyrus, inferior occipital gyrus | hOc4v, Lobule VIIa crusI, hOc3v | L | -26 | -87 | --18 | 5.14 | 67 | 0.403 |
| *Boys < girls* | | | | | | | | |
| Cerebellar hemisphere, fusiform gyrus, parahippocampal gyrus, lingual gyrus, subiculum | Lobule I-V | L | -12 | -37 | -14 | 5.25 | 270 | 0.343 |
